# How Do Algorithmic Management Practices Affect Workforce Well-Being? A Parallel Moderated Mediation Model

**DOI:** 10.3390/bs14121123

**Published:** 2024-11-23

**Authors:** Husam Zayid, Ahmad Alzubi, Ayşen Berberoğlu, Amir Khadem

**Affiliations:** Department of Business Administration, Institute of Graduate Research and Studies, University of Mediterranean Karpasia, 33010 Mersin, Turkey; 200634014@std.akun.edu.tr (H.Z.); aysen.berberoglu@akun.edu.tr (A.B.); amir.khadem@akun.edu.tr (A.K.)

**Keywords:** algorithmic management, workforce well-being, job burnout, person–job fit, perceived threat

## Abstract

Modern workplaces increasingly use algorithmic management practices (AMPs), which shape task assignment, monitoring, and evaluation. Despite the potential benefits these practices offer, like increased efficiency and objectivity, their impact on workforce well-being (WFW) has raised concerns. Drawing on self-determination theory (SDT) and conservation of resources theory (COR), this study examines the relationship between algorithmic management practices and workforce well-being, incorporating job burnout (JBO) and perceived threat (PT) as parallel mediators and person–job fit (PJF) as a moderator. The research employed a cross-sectional survey design targeting 2450 KOSGEB-registered manufacturing SMEs in Istanbul, Turkey. A sample of 666 respondents participated, and the data were analyzed using Smart PLS 4, employing structural equation modeling to test the proposed model. The results indicated that algorithmic management practices significantly increased job burnout and perceived threat, both of which negatively impacted workforce well-being. However, the direct effect of algorithmic management practices on workforce well-being was non-significant. Person–job fit moderated the relationships between algorithmic management practices and both job burnout and perceived threat, further influencing workforce well-being. The findings underscore the critical need for organizations to balance algorithmic efficiency with human-centric practices. Prioritizing person–job fit and fostering transparency in algorithmic processes can mitigate negative impacts, enhance employee well-being, and drive sustainable organizational success in the digital age.

## 1. Introduction

Technological advancements, at the forefront of Industry 4.0, have brought about profound changes across various facets of human society [1,2], including the management of businesses and organizational structures [3]. In recent years, researchers have observed a growing interest in and concern for the impact of technological innovations on human resource management [4], with particular attention to algorithmic management practices, which are considered one of the most disruptive forms of technological change currently underway [5,6].

Initially, the term algorithmic management practices described how gig-working platforms utilized sophisticated algorithms to allocate, optimize, and evaluate work [7]. While these traits are typical of algorithmic management practices, they offer a restrictive definition that excludes many algorithmic practices currently implemented in workplaces [8]. For instance, in Amazon, algorithms are used extensively to manage and optimize various aspects of operation to determine the most efficient routes for workers to pick items from shelves, ensuring that they can fulfill orders as quickly as possible. Job burnout affects employees, with contributing factors such as limited career growth opportunities and inadequate management support. Such an environment encourages employees to seek employment in different fields or organizations [9].

Conversely, higher organizational commitment and job satisfaction are associated with lower levels of burnout [10]. While developing management algorithms aims to improve organizational efficiency and individual productivity, new challenges arise for both organizations and their employees. The reliance on algorithms can lead to the dehumanization of human resource management, compromising the personal and empathetic aspects of managing people [11]. Further analysis and research are needed to understand the impact of these practices on employee well-being.

Job burnout is a critical factor influencing various work outcomes [12]. Algorithmic management practices alter how work is performed, reducing personal influence in the process. Studies suggest a negative relationship between job burnout and control over work, particularly in highly skilled positions [13]. These effects can diminish job burnout and responsibility within tasks, potentially affecting workforce well-being [14]. Existing research lacks sufficient empirical analysis of the positive or negative effects of algorithmic management practices on workforce well-being, both directly and indirectly, through their impact on work autonomy and overall compensation [15]. The study seeks to answer the following research questions:

RQ 1: How do algorithmic management practices affect workforce well-being and what roles do perceived threat and job burnout play in mediating this relationship?

RQ 2: To what extent does a person’s job fit to play as a moderator influence algorithmic management practices and workforce well-being?

RQ 3: What strategies can organizations implement to mitigate the negative effects of algorithmic management practices?

This study adds to the ongoing conversation by combining the theoretical frameworks of self-determination theory and conservation of resources theory. To examine the relationship between algorithmic management practices and workforce well-being, we incorporated job burnout and perceived threat as parallel mediators and person–job fit as a moderator. We achieved this by using a large dataset from many different organizations. This study contributes to the literature by providing valuable insights into how algorithmic management practices are often utilized to enhance employee mental health and satisfaction. While previous studies have primarily focused on the benefits of algorithmic management practices for productivity and organizational control, fewer have examined their implications for workforce well-being. By introducing perceived threat and job burnout as mediating factors, this study identifies the specific pathway through algorithmic management practices that may negatively impact workforce well-being. Additionally, examining person–job fit as a moderator highlights the individual difference in response to algorithmic management practices which could inform tailored management strategies.

Despite the growing adoption of algorithmic management practices across industries, the well-being-related consequences for employees remain unexplored; existing studies [6] largely examine algorithmic management practices in terms of performance outcomes with limited focus on job burnout and perceived threat as mediators and person–job fit as a moderator. This study addresses this gap by focusing on the workforce well-being of algorithmic management practices and testing how individual factors can potentially exacerbate these effects using cross-sectional data, and it endeavors to bridge this gap. The research is structured into three sections: (1) theoretical hypothesis development, elucidating the essence and research models of workforce well-being, algorithmic management practices, job burnout, and perceived threat; (2) a methodology section detailing the empirical research approach, including the research objectives, assumptions, sample characteristics, and results of statistical analyses; and (3) a discussion section culminating in conclusions.

## 2. Theoretical Background and Hypotheses Development

### 2.1. Self-Determination Theory

Psychologists Edward Deci and Richard Ryan first introduced their ideas in their 1985 book, Self-Determination and Intrinsic Motivation in Human Behavior. Self-determination theory represents a broad theory of human motivation that has found successful application across various domains and management [16]. It asserts that an employee’s motivational orientation towards job activities influences both their performance and well-being. Self-determination theory categorizes motivation into different types and suggests that these types have distinct antecedents, relations to conservation of resources theory, and outcomes.

Over the years, self-determination theory has addressed the intersection of motivation with organizational performance and wellness concerns, emphasizing the importance of fostering sustainable motivation and volitional engagement among employees and customers [17]. Self-determination theory advocates for creating workplace environments that support employees’ autonomy, not only as an end in itself but also as a means to enhance employee satisfaction, well-being, and organizational effectiveness.

Various factors, such as managerial styles and compensation structures, which contribute to employee autonomy and competence at work, are essential components for fostering engagement and organizational success, as outlined by self-determination theory. Self-determination theory underscores how managerial actions significantly shape organizational factors, either supporting or hindering employees’ fundamental psychological needs.

The fulfillment of all three basic psychological needs often strongly relates to conservation of resources theory and organizational support for autonomy [18,19]. This conservation of resources theory relation arises because managers who encourage autonomy tend to be responsive to other needs as well, and employees with autonomy are more likely to actively meet their additional psychological needs. Therefore, when employees feel supported in their autonomy, they typically develop a stronger connection to the organization and perceive themselves as more capable and effective [10,16].

### 2.2. Conservation of Resources Theory

Stevan Hobfoll introduced conservation of resources theory in the 1980s. This theory is a stress theory that arises when essential resources become endangered, depleted, or unattainable despite significant efforts. Conservation of resources theory states that individuals actively seek to acquire, retain, nurture, and safeguard resources they consider essential. There is an understanding that cognitive processes inherently prioritize resource loss over resource gain, which stems from evolutionary imperatives [20].

Fundamentally, conservation of resources theory serves as a motivational framework grounded in the evolutionary drive to secure and maintain essential resources for survival, and conservation of resources theory is an element of human behavioral genetics. People use resources not only to cope with stress but also to accumulate a reserve for future adversity. The acquisition and retention of personal, social, and material resources instill in individuals, families, and organizations a sense of capability to navigate stressful situations [21].

Conservation of resources theory emphasizes that individual appraisal of stress is secondary to the central and universal value placed on resources such as health, well-being, family, self-esteem, and purpose in life. While the cultural expressions of these appraisals may differ, they consistently reflect this conservation of resource elements [22]. Moreover, conservation of resources theory emphasizes that we should not analyze stress solely as discrete events, but rather as complex sequences that unfold over time.

For instance, in a workplace experiencing layoffs, anticipation precedes the actual event, followed by the process of adjustment or finding new employment. Objective factors such as employability, skills, financial savings, and the availability of alternative positions significantly shape outcomes. Individuals’ appraisals, which are generally favorable predictors, stem from their innate tendency to prioritize resource loss over gain, as emphasized by conservation of resources theory [20]. This bias is culturally pervasive and not unique to individuals, although personal experiences may influence such appraisals.

The autonomous use of data-driven artificial intelligence is referred to as algorithmic management practices. While it can improve certain aspects of decision-making, it may also impact job autonomy, perceived fairness, and overall well-being. Existing research indicates that algorithmic management practices exert both direct and indirect effects on well-being. Empirical findings suggest a moderate, direct influence of algorithmic management practices on workforce well-being. Organizations adopting conservation of resources theory principles can positively influence employee well-being.

### 2.3. Algorithmic Management Practices and Workforce Well-Being

Algorithmic management practices, which involve autonomous decision-making in human resource management using self-learning algorithms and AI, can have a significant impact on employees’ well-being in the workplace. One emerging trend is the adoption of algorithmic management practices, characterized by the integration of algorithms in managerial tasks encompassing HRM and decision-making processes [23], which has introduced new paradigms.

We first coined the term “algorithmic, data-driven management” to describe innovative methods for task assignment, informational support, and performance evaluation, specifically within the ridesharing sector. An algorithm operates as a computational tool that autonomously makes decisions using statistical models or decision rules, eliminating the need for direct human intervention [24]. Algorithmic management practices are defined as a control system where self-learning algorithms take on the responsibility for making and implementing decisions that impact labor, thereby reducing human oversight and involvement in the labor process. This results in the automation of HR-related tasks and duties, previously managed by human personnel [25].

In terms of workforce well-being, the employer, as the key architect of the work environment, has a significant role. The literature provides specific recommendations for fostering a supportive and effective workplace, including ensuring employees receive living wages, have autonomy in their work, access professional development opportunities, and are accommodated for disabilities with facilitated return-to-work programs [6,26]. Studies indicate that algorithmic management practices alter work dynamics and diminish personal influence over processes. There exists a negative conservation of resources theory relation between job burnout and a heightened sense of control over work, especially in highly skilled positions.

While algorithms can positively impact decision-making concerning personnel, they may also detrimentally affect job burnout, perceived threat, and overall workforce well-being. Algorithmic management practices exert a direct influence on workforce well-being. Larger organizations tend to experience diminishing impacts of algorithmic management practices on well-being and job burnout. While algorithmic management practices have the potential to improve decision-making, organizations must carefully consider their repercussions on employee autonomy and overall well-being. Striking a balance between automation and human agency is imperative for fostering a positive work environment. Therefore, the hypothesis put forward is as follows:

**H1.**  *Algorithmic management practices negatively influence workforce well-being.*

### 2.4. Algorithmic Management Practices and Influences on Job Burnout

Algorithmic management practices refer to the use of algorithms and AI to manage work processes, tasks, and employee performance. They have proliferated across diverse industries owing to technological advancements. The extant literature on job burnout delineates three components, which constitute the subscales of the Maslach Burnout Inventory [26]. Exhaustion, the most studied dimension, signifies stress aspects, including intense physical and emotional fatigue that impedes efficient functioning.

Professional inefficacy, or reduced personal accomplishment, refers to negative self-evaluation and a profound sense of ineffectiveness in work and interpersonal relationships. However, research indicates that burnout’s conservation of resources theory dimensions are exhaustion and cynicism, or disengagement. This section aims to provide an overview of job burnout, its causes, and its repercussions on individuals and organizations. Additionally, it elucidates individual and organizational interventions to prevent or alleviate burnout severity [27].

We also examine the theoretical perspective of algorithmic management practices on worker well-being. Self-determination theory posits that individuals possess intrinsic psychological needs that enhance motivation and well-being. Algorithmic management practices’ effects on worker motivation are often negative, as algorithmic systems’ rigidity may restrict autonomy and relatedness. However, person–job fit usage practices can moderate these effects. For instance, when algorithmic management allows some autonomy or facilitates clear communication, its impact on motivation may vary.

Organizations should integrate self-determination theory principles into the design and implementation of algorithmic management practices systems, balancing algorithmic control with opportunities for autonomy and relatedness to enhance worker motivation and well-being [28]. Conservation of resources theory underscores how individuals endeavor to acquire, safeguard, and sustain resources to handle stressors. Prolonged work-related stress leads to job burnout, a condition characterized by emotional fatigue, depersonalization, and diminished personal achievement. Frequently, this is due to resource depletion [29]. Algorithmic management practices can influence resource availability, impacting burnout risk. In essence, understanding the interplay between algorithmic management practices, worker motivation, and job burnout necessitates consideration of theories such as self-determination theory and conservation of resources theory. Organizations must strike a balance between algorithmic control and human needs to foster workforce well-being and productivity. Accordingly, the following is hypothesized:

**H2.**  *Algorithmic management practices positively influence job burnout.*

### 2.5. Job Burnout and Workforce Well-Being

Anxiety, sickness, despondency, and exhaustion represent facets of diminished mental health and overall well-being in individuals. Similarly, headaches and muscle pains serve as indicators of physical unwellness. Employee workforce well-being catalyzes organizational success, shielding the organization from decreased productivity and reducing costs associated with poor health insurance.

Forward-thinking organizations must ensure that their initiatives yield health outcomes that contribute to overall workforce well-being, with a focus on the physical workspace and organizational culture. JBO results from chronic workplace stress that goes unmanaged. It is characterized by emotional exhaustion, depersonalization, and reduced feelings of accomplishment. Burnout can lead to a decline in an employee’s well-being and can also have negative effects on their physical health [30].

Substantial evidence from previous studies highlights a significant relationship between workforce well-being and job burnout [31,32,33,34,35]. Engaged employees are characterized as motivated, self-directed, and valuable contributors who drive organizational growth and development [36]. A positive state of mind is characterized by vigor, dedication, and absorption. Engaged employees feel energetic and effective in their work, and they are able to deal with the demands of their jobs [37]. The existing literature suggests that adverse workplace conditions negatively affect individual involvement, job satisfaction, and work enthusiasm, while also harming employee well-being [38]. According to the conservation of resources theory, there is a negative association between job burnout and workplace factors [39]. Consequently, job burnout tends to be more pronounced in unfavorable work environments. Based on the aforementioned discussion, the following hypothesis is formulated:

**H3.**  *Job burnout has a negative influence on workforce well-being.*

### 2.6. Algorithmic Management Practices and Perceived Threat

Algorithmic management practices involve using algorithms, data analytics, and automated decision-making systems to manage and control work processes and employees. These practices can include automated scheduling, performance tracking, task allocation, and feedback systems. Perceived threat refers to the individual’s subjective assessment of potential harm or danger to their well-being, status, or resources. In the workplace, perceived threats can manifest as fears related to job security, performance evaluations, and personal autonomy [23,40].

The hypothesis posits that implementing algorithmic management practices in the workplace can improve perceived threats to employees’ performance. This hypothesis suggests that reliance on automated systems for management decisions can make employees feel more vulnerable, examined, and insecure about their job stability and autonomy [19]. Self-determination theory was developed, positing that the need to satisfy three fundamental psychological needs drives human motivation and well-being. Algorithmic management practices can undermine employees’ sense of autonomy by rigidly controlling work schedules, task assignments, and performance evaluations. This lack of control can lead to feelings of frustration and helplessness.

The conservation of resources theory, proposed by [22], suggests that individuals strive to obtain their valuable resources, which can be personal. Algorithmic management practices can create a perceived threat to employees’ job security and stability, as decisions may seem arbitrary or opaque, increasing anxiety about potential job loss. Continuous monitoring and automated evaluations can lead to emotional exhaustion and stress, depleting personal resources like energy and self-esteem. Algorithmic systems may undervalue or improperly recognize employees’ efforts, leading to decreased motivation and a reduced perceived return on their work investments [41].

Self-determination theory’s emphasis lies on meeting employees’ psychological needs and how their undermining by algorithmic management practices can increase perceived threat. Conservation of resources theory highlights the role of resource management, where algorithmic management practices can threaten or deplete valuable resources, leading to increased stress and perceived threat [42]. By fulfilling the psychological needs outlined in self-determination theory and protecting the resources highlighted in conservation of resources theory, organizations can reduce the perceived threat associated with algorithmic management practices and promote a healthier, more supportive work environment. Drawing from the theoretical framework, the hypothesis is outlined as follows:

**H4.**  *Algorithmic management practices positively influence perceived threat.*

### 2.7. Perceived Threat and Workforce Well-Being

Perceived threat refers to an individual’s subjective evaluation of the likelihood and severity of potential harm or negative outcomes in a given situation. External events or various factors can influence this, posing a risk to one’s job or personal well-being [43]. Workforce well-being encompasses the overall mental, emotional, and physical health of employees within an organization. It includes factors of fulfillment in the workplace [44].

The hypothesis is that perceived threat negatively influences workforce well-being. This means that when employees feel threatened, whether by job insecurity, organizational changes, or external factors, they are likely to experience a decline in their overall well-being. This decline can manifest as increased stress, lower job satisfaction, reduced motivation, and overall poorer mental and physical health [45].

Self-determination theory posits that when employees perceive a threat, such as the potential loss of their job or drastic changes in their work environment, their sense of autonomy and competence can be undermined [46]. They may feel less in control of their future and less capable of performing their job effectively under the looming threat. Additionally, the stress and anxiety caused by perceived threat can harm their relationships with colleagues, further impacting their relatedness needs. The frustration of these basic psychological needs can lead to decreased well-being [47].

Conservation of resources theory suggests that individuals strive to protect their resources. Stress occurs when there is potential for actual resource loss or insufficient resource acquisition despite significant investment [48]. Perceived threats in the workplace represent a potential loss of valuable resources, such as job security, income, professional status, and social support. The anticipation or experience of these losses can deplete employees’ resources, leading to heightened stress and anxiety.

As employees invest more effort to cope with or prevent these losses, they may experience burnout and diminished well-being [46]. This continuous state of resource depletion and the associated stress can severely impact their mental and physical health. We can understand the negative impact of perceived threats on workforce well-being by integrating self-determination theory and conservation of resources theory. According to self-determination theory, perceived threats undermine employees’ basic psychological needs, leading to reduced well-being [32,33]. Conservation of resources theory complements this by explaining that the anticipation or experience of resource loss due to perceived threats further depletes employees’ resources, exacerbating stress and reducing overall well-being. Together, these theories provide a comprehensive framework for understanding how perceived threats can negatively impact workforce well-being by disrupting psychological needs and depleting valuable resources. Given the preceding arguments, the proposed hypothesis is stated as follows:

**H5.**  *Perceived threat negatively influences workforce well-being.*

### 2.8. Job Burnout as a Mediator

The term algorithmic management practices refers to the use of sophisticated algorithms to monitor, evaluate, and manage employees [49]. This includes practices like automated scheduling, performance tracking, and behavior monitoring. Such practices can lead to increased efficiency but can also result in increased pressure and stress for employees. Job burnout is a state of job stress. It manifests as sensations of fatigue and diminished professional effectiveness [42]. Workforce well-being encompasses various aspects of employees’ health and happiness [44]. This hypothesis suggests that job burnout mediates the negative impact of algorithmic management practices on workforce well-being. In other words, algorithmic management practices increase job burnout, which in turn reduces workforce well-being.

Algorithmic management practices often reduce employees’ sense of autonomy and relatedness by imposing rigid controls and constant monitoring. This can lead to a lack of fulfillment of these basic psychological needs, increasing stress and burnout. Consequently, job burnout mediates the relationship between algorithmic management practices and workforce well-being. Job burnout and workforce well-being have a complex relationship [50]. There is also research that shows the positive impact on algorithms while offering employees the freedom to create value [50]. This means that, despite the use of algorithmic management, employees may have some freedom to feel job burnout. Also, [49] showed that algorithmic management practices can lead to resource loss by increasing job demands without providing adequate support or resources. This resource depletion can lead to job burnout, which in turn negatively affects workforce well-being. Thus, job burnout acts as a mediator in the relationship between algorithmic management practices and workforce well-being.

By integrating self-determination theory and conservation of resources theory, we can understand the mediating role of job burnout in the relationship between algorithmic management practices and workforce well-being. Algorithmic management practices often undermine employees’ fundamental psychological needs and result in resource depletion. This combination boosts job burnout, which hurts workforce well-being. In summary, job burnout mediates the negative impact of algorithmic management practices on workforce well-being, as these practices undermine essential psychological needs and deplete valuable resources, resulting in increased stress and reduced well-being. In light of the evidence discussed, the hypothesis can be articulated as follows:

**H6.**  *Algorithmic management negatively influences workforce well-being through the negative influence of job burnout.*

### 2.9. Perceived Threat as a Mediator

Perceived threat refers to the subjective assessment of potential harm or danger arising from a situation or environment. This can include concerns about job security in the workplace [50]. As previously defined, algorithmic management practices involve using algorithms to oversee and supervise various aspects of organizational processes. These practices can lead to heightened efficiency but may also cause employees to feel constantly monitored and evaluated [25]. This hypothesis suggests that the perceived threat mediates the negative impact of algorithmic management practices on workforce well-being. In other words, algorithmic management practices increase perceived threat, which in turn reduces workforce well-being. Algorithmic management practices can undermine employees’ sense of autonomy and relatedness. This can result in employees perceiving a threat to their autonomy and job security. The perceived threat response to these psychological needs can increase stress and reduce workforce well-being, making it a mediator in the relationship between algorithmic management practices and workforce well-being.

By increasing job demands and reducing employees’ control over their work environment, algorithmic management practices can lead to perceived threats. This perception of threat can result in resource loss, leading to increased stress and reduced well-being [51]. Thus, perceived threat acts as a mediator in the relationship between algorithmic management practices and workforce well-being. By integrating self-determination theory and conservation of resources theory, we can understand the mediating role of perceived threat in the relationship between algorithmic management practices and workforce well-being. Algorithmic management practices often undermine employees’ basic psychological needs and create a perception of a threat to their resources. This combination increases perceived threat, which negatively impacts workforce well-being. In summary, perceived threat mediates the negative impact of algorithmic management practices on workforce well-being, as these practices undermine essential psychological needs and create a perception of threat to valuable resources, resulting in increased stress and reduced well-being. Therefore, the hypothesis suggested by this review is as follows:

**H7.**  *Algorithmic management practices negatively influence workforce well-being through the negative influence of perceived threats.*

### 2.10. Person–Job Fit as a Moderator

Person–job fit refers to the compatibility between an individual’s abilities and desires, as well as the demands, rewards, and attributes of a particular job. A high person–job fit means that the job characteristics align well with the individual’s skills and preferences, leading to greater job satisfaction and performance [52]. Person–job fit moderates the relationship between algorithmic management practices and job burnout. Particularly, the positive association between algorithmic management practices and job burnout diminishes when employees possess a greater level of person–job fit. Person–job fit also moderates the connection between algorithmic management practices and perceived threat, specifically weakening the positive conservation of resources theory relation between algorithmic management practices and person–job fit when employees demonstrate a higher degree of person–job fit [53].

Furthermore, person–job fit moderates the link between algorithmic management practices and workforce well-being. In particular, the negative association between algorithmic management practices and workforce well-being is less pronounced among employees with a higher level of person–job fit. Person–job fit denotes the alignment between an individual’s competencies, skills, and preferences and the demands of a specific job role. When this alignment is optimal, employees typically experience increased job satisfaction, improved performance, and increased retention rates. On the other hand, poor fit can lead to dissatisfaction, stress, and turnover. Organizations assess person–job fit through job analysis, personality assessments, interviews, and evaluations. In the SME industry, achieving a good person–job fit is crucial for employees who directly interact with customers, as it positively impacts the customer experience [50,54]. AI can help identify suitable candidates during recruitment and optimize job assignments.

By applying self-determination theory and conservation of resources theory, we can understand how person–job fit moderates the impact of algorithmic management practices on job burnout, perceived threat, and workforce well-being. A high person–job fit enhances employees’ sense of autonomy (self-determination theory) while also providing valuable resources (conservation of resources theory). This reduces the stress and burnout associated with algorithmic management practices. A favorable fit mitigates threat perception by making employees feel secure and competent (self-determination theory) and less likely to experience resource loss (conservation of resources theory). Employees with a high person–job fit are better able to satisfy their psychological needs (self-determination theory) and conserve their resources (conservation of resources theory), ensuring their well-being even in the presence of algorithmic management practices. In summary, person–job fit serves as a critical moderator that can mitigate the negative impacts of algorithmic management practices on job burnout, perceived threat, and workforce well-being by fulfilling basic psychological needs and conserving essential resources. Building on the insights provided, the hypotheses are defined as follows:

**H8.**  *Person–job fit moderates the relationship between algorithmic management practices and job burnout, and the positive relationship becomes weaker (stronger) when employees have a higher (lower) degree of person–job fit in the workplace.*

**H9.**  *Person–job fit moderates the relationship between algorithmic management practices and perceived threat, and the positive relationship becomes weaker (stronger) when employees have a higher (lower) degree of person–job fit in the workplace.*

**H10.**  *Person–job fit moderates the relationship between algorithmic management practices and workforce well-being, and the negative relationship is weaker among employees with a higher degree of person–job fit compared to a lower degree of job fit in the workplace.*

**Figure 1:**
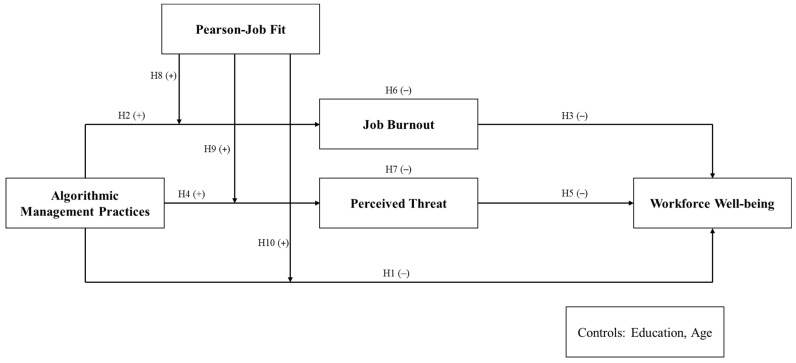
Research model.

The relationships among algorithmic management practices, job burnout, perceived threat, workforce well-being, and the moderating role of person–job fit are synthesized into the proposed research model. This model visually represents the hypothesized connections and provides a framework for understanding the study’s conservation of resources theory and variables, as illustrated in Figure 1.

## 3. Methodology

### 3.1. Sample and Data Collection

To ensure the representativeness of the sample, employees from small- and medium-sized businesses (SMEs) in Turkey registered with the Turkish Small Business Administration (KOSGEB) were recruited for participation in the study. Given the lack of consensus on the definition of SMEs, particularly across different countries and sectors, this study adopted the criterion of employing fewer than 250 employees, consistent with both the Turkish Small Business Administration and the Turkish State Institute of Statistics. Moreover, a minimum threshold of at least 10 employees was set to exclude micro firms, aligning with established small business research practices [55].

The SME sector is central to Turkey’s economy, driving job creation, economic stability, and innovation. Representing over 99% of enterprises, SMEs generate approximately 73% of employment and contribute around 54% to the GDP [56]. The manufacturing industries selected for inclusion encompassed a diverse array of sectors, ranging from food and beverage to chemical and petrochemical, ensuring a comprehensive representation of the Turkish manufacturing landscape. However, their environmental impact is significant, particularly in manufacturing, where they contribute notably to industrial emissions and resource consumption [57]. Recent policies encourage sustainable practices, though challenges persist in balancing growth with environmental goals. SMEs’ influence in Turkey highlights their role in both economic development and sustainable progress. The study targeted SMEs operating in the manufacturing sector within the greater metropolitan area of Istanbul, Turkey. This choice was informed by Istanbul’s historical prominence as the country’s primary industrial and commercial hub, accounting for a substantial portion of total capital investment and GDP.

In manufacturing, AMPs facilitate workforce performance monitoring; automated shift scheduling; and optimized resource allocation in logistics, storage, and delivery [58]. AMPs coordinate tasks, track employee movements through wearables, and provide real-time data for informed managerial decisions [59]. While AMPs are more established in retail and food services, where algorithmic shift scheduling is prevalent [60,61], their potential to boost operational efficiency in manufacturing is increasingly recognized. The trend reflects a broader shift towards digital technologies in workforce management, with AI-based recruitment and monitoring tools indicating a future where AMPs may become standard across industries [25,62]. AMPs’ integration into manufacturing thus signals a shift toward data-driven methods that reshape traditional production and workforce management.

Data collection was conducted through the distribution of self-administered questionnaires to executives of 2450 KOSGEB-registered manufacturing SMEs within the designated area. Data were collocted between June and July 2024, and executives were personally contacted to elucidate the study’s objectives and seek their approval for participation [63]. Upon obtaining management consent, employees were contacted via email, outlining the survey’s purpose, emphasizing voluntary participation, and ensuring confidentiality. This approach aimed to foster a conducive environment for candid responses while respecting participants’ autonomy and privacy.

To ensure rigor and transparency in our sampling process, we adhered to ethical standards, obtaining informed consent from all participants by explaining the study’s purpose, procedures, and voluntary nature. Using purposive sampling, we targeted professionals in the manufacturing sector to capture relevant insights on AMPs and workplace dynamics.

A total of 666 individuals completed the questionnaires, yielding a response rate of 27.18% from the targeted participant pool. This response rate, while modest, reflects a substantial level of engagement and provides a robust dataset for analysis. Additionally, participants were assured of the opportunity to receive feedback on the research outcomes as a token of appreciation for their cooperation, further incentivizing their involvement.

### 3.2. Basic Characteristics of the Respondent Profile

The respondent profile in this study reflects a diverse array of small- and medium-sized enterprises (SMEs) operating within the manufacturing sector of Turkey (Table 1). Across various industry segments, SMEs specializing in food products, electrical equipment, and chemical products emerged as the most prominent, contributing 160, 115, and 102 respondents, respectively. Additionally, significant representation was observed from machinery and equipment (93), rubber and plastic (67), textiles (55), and pharmaceutical sectors (43), with an unspecified “other” manufacturing subgroup comprising 31 respondents. The workforce within these companies was predominantly male, comprising 60.2% of the sample, with individuals aged 41–50 representing the largest subset at 41.3%. In terms of business longevity, a substantial portion of participants (206) had operated between 6 and less than 10 years, while 156 respondents had businesses operational for less than 5 years. Moreover, 125 establishments had operated for more than 11 but less than 15 years, 110 for more than 16 but less than 20 years, and 69 businesses boasted operational histories exceeding 20 years. The majority of respondents identified their businesses as medium-sized enterprises (less than 250 employees), constituting approximately 57.8% of the sample, followed by small businesses (less than 50 employees) at 42.2%.

### 3.3. Measurement of Variables

The measures employed to capture data for the empirical analyses drew upon established questionnaires utilized in prior studies (Appendix A), ensuring the reliability and validity of the instruments. Given the adaptation of the survey instrument for use in Turkey, the translating-backward translation methodology was adopted to facilitate linguistic equivalence and cultural appropriateness. Moreover, the questionnaire underwent rigorous pre-testing iterations to validate the sequencing of questions, wording, and format, thereby enhancing the instrument’s comprehensibility and clarity. Participants rated their agreement with each item on a 5-point Likert scale, ranging from strongly disagree (1) to strongly agree (5).

The study assessed respondents’ perceptions of AMPs using a four-item scale adapted from [6,64]. This scale covered dimensions such as the utilization of data analytics for monitoring employee performance, enhancing processes or service delivery through data analytics, the adoption of robots, and the establishment of work pace by machines or computers.

JBO, a crucial aspect of workforce well-being, was evaluated using a nine-item scale adapted from previous research [65,66]. This comprehensive scale assessed various dimensions of burnout and reduced personal accomplishment. By considering these facets, researchers aimed to holistically evaluate participants’ experiences related to burnout [67].

Perceived threat, another salient variable in the study, was measured using a four-item scale adapted from [68,69]. This scale captured respondents’ subjective perceptions of threat arising from AMPs, providing insights into their cognitive appraisal of potential risks and challenges.

The moderating role of PJF was assessed using a six-item scale adapted from [70], which encompassed dimensions such as skills–requirements fit, needs–supplies fit, and values–congruence fit. This scale enabled an exploration of the extent to which individuals’ alignment with their job roles moderates the relationship between AMPs and workforce well-being.

Finally, workplace well-being, the dependent variable in the model, was measured using an eight-item scale adapted from [71]. This scale captured various dimensions of well-being, including physical health, psychological well-being, job satisfaction, and work–life balance, offering a comprehensive assessment of participants’ overall well-being within the workplace context.

### 3.4. Statistical and Analytical Strategy

To analyze the empirical data collected from the survey and examine the complex inter-relationships among variables, a structural equation modeling (SEM) approach was employed, utilizing Smart PLS 4 as the analytical tool [72]. Following the methodological guidelines proposed by [73], a two-stage analysis strategy was implemented. In the initial stage, attention was directed towards evaluation of the measurement model; researchers assessed the reliability and validity of the measurement instruments used to operationalize the constructs under investigation. This process involved examining factors such as convergent validity, discriminant validity, and internal consistency reliability to ensure the robustness of the measurement model [74]. In the subsequent stage, the structural model was tested to scrutinize its fit and relationships among variables and ascertain the underlying mechanisms driving these associations. As emphasized by [75], SEM offers a unified framework for assessing both direct and indirect effects, thus facilitating a comprehensive exploration of the complex pathways underlying the phenomena of interest. This analytical approach enables a nuanced understanding of the intricate interplay between AMPs, individual perceptions, and workforce well-being, thereby advancing theoretical discourse and informing evidence-based interventions aimed at enhancing organizational effectiveness and employee welfare.

## 4. Analysis and Results

### 4.1. Robustness Check

Prior to delving into the substantive analysis, preliminary assessments were conducted to ensure the suitability of the data for structural equation modeling (SEM). This involved scrutinizing the descriptive statistics, reliability, and validity of the measurement model to ascertain the robustness of the dataset. As highlighted by [76], one of the crucial considerations in multivariate analysis is the presence of normality in the data distribution, as deviations from normality can compromise the validity and reliability of findings. To this end, skewness and kurtosis values were examined, with no variable exhibiting skewness greater than 3 or kurtosis values exceeding 10 [77], indicating adherence to normality assumptions. Additionally, potential multicollinearity issues were evaluated through the examination of variance inflation factor (VIF) values, with all VIF values falling within the acceptable range of 1.467 to 4.386, well below the threshold maximum value of 10 [75]. These preliminary assessments affirm the appropriateness of the dataset for SEM analysis, laying the groundwork for subsequent substantive analyses aimed at unraveling the complex relationships among variables.

### 4.2. Common Method Variance

To mitigate the potential issue of common method variance, we implemented procedural and statistical remedies as recommended by reference [78]. These measures were particularly important since data were collected at a single time point. Procedurally, measures were taken to enhance respondent anonymity and encourage candid responses by emphasizing the absence of correct or incorrect answers and urging openness. Furthermore, efforts were made to articulate the study’s purpose clearly, minimize priming effects through careful item arrangement, and reduce question ambiguity via pilot testing [79]. These procedural safeguards aimed to mitigate methodological biases and enhance data quality. Additionally, as a statistical remedy in the form of addressing the potential issue of common method variance, we utilized the Harman single-factor procedure. According to reference [80], if common method variance is prevalent, a single component would account for a majority of the data variance. However, the results from our study revealed that the first extracted component explained only 34% of the variation. This suggests that common method variance is not a substantial concern in our research. These combined procedural and statistical measures enhance confidence in the validity and reliability of the data, providing a solid foundation for subsequent analyses and interpretations.

### 4.3. Validity and Reliability of the Measurement Model

**Figure 2:**
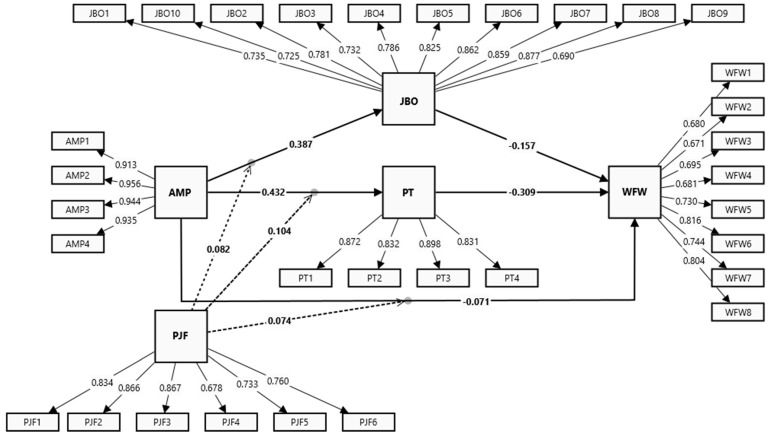
Evaluation of the measurement model. Note: Dashed arrows represent moderating effect, sold arrows represent direct relationships.

Prior to assessing the inter-relationships between constructs, a comprehensive evaluation of the measurement model was conducted to ensure its validity and reliability. Following the guidelines outlined by [81], confirmatory composite analysis (CCA) was employed to validate the hypothesized five-factor model, encompassing AMP, JBO, PT, PJF, and WFW. As depicted in Figure 2, outer-loading values exceeded the recommended threshold of 0.60 [73], which underscores the robustness of the measurement model. Additionally, all item component loadings were statistically significant (*p* < 0.001) and ranged from 0.671 to 0.956, affirming convergent validity [72].

Descriptive statistics presented in Table 2, including mean values and standard deviations, offer insights into the distribution and variability of responses across constructs. CR values for all constructs ranged from 0.901 to 0.966, exceeding the threshold of 0.70. This indicates strong internal consistency reliability. AVE values ranged from 0.532 to 0.878, surpassing the predetermined criterion of 0.50. Importantly, the CR scores for all constructs exceeded their respective AVE values (CR > AVE), providing further evidence of convergent validity (Table 2). Discriminant validity was assessed by comparing the square root of AVE values with inter-construct correlation values [82]. These robust measurement model results lay a solid foundation for subsequent analyses and interpretations.

In evaluating CR for our measurement scales, we observed some CR values exceeding 0.95, which Hair et al. [74] suggest could indicate item redundancy. However, this outcome aligns with research showing that trait-oriented scales—such as those measuring AMP and job burnout—typically exhibit higher reliability due to their temporal stability, unlike state-oriented measures [63]. The trait-based nature of AMPs and job burnout thus explains the elevated CR values, which indicate strong internal consistency rather than redundancy. These results affirm the reliability of our trait constructs, consistent with psychometric expectations and the study’s objectives.

Regarding discriminant validity, the square root of AVE values exceeded correlation values with other constructs [82]. In Table 3, the square root of AVE values for each construct surpassed correlation values with other constructs, affirming the measurement scales’ ability to accurately differentiate between constructs.

To further confirm discriminant validity, we used the Heterotrait–Monotrait (HTMT) ratio as recommended by Henseler et al. [83], given its increased sensitivity in detecting discriminant validity issues in PLS-SEM models. HTMT values are presented in Table 3, all of which meet the established threshold of 0.85, supporting discriminant validity across constructs [84]. Together with the Fornell–Larcker criterion, these HTMT results enhance the robustness of our measurement model, indicating that our constructs are distinct and reliable for accurately capturing the conceptual boundaries needed for analyzing variable inter-relationships.

### 4.4. Direct Effects Estimations

SEM was utilized to examine both the direct and indirect relationships among the study variables. This approach provides insights into the intricate pathways that underlie the impact of AMP on WFW in SMEs. After parameter estimation, bootstrapping was conducted to validate the robustness of the findings with 5000 Bootstrap samples generated through resampling with replacement from the original dataset [85]. Remarkably, the coefficients estimated through bootstrapping closely aligned with those derived from Smart PLS 4, affirming the reliability of the results [72].

**Figure 3:**
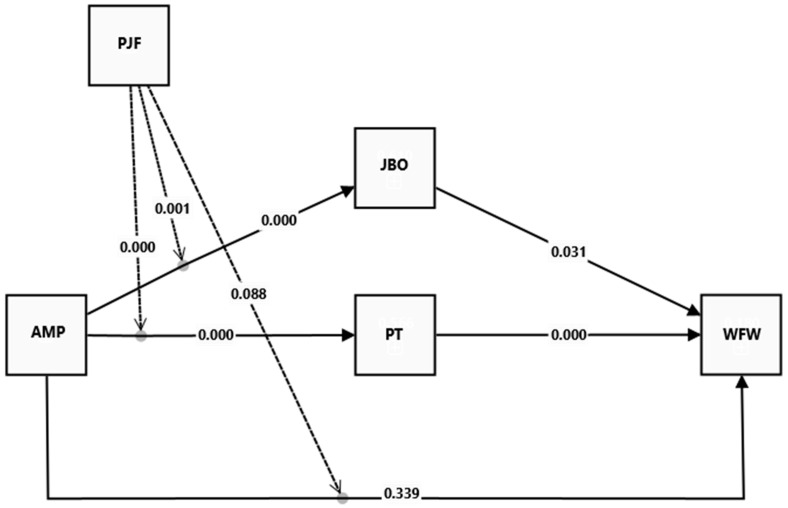
Estimation of the structural model. Note(s): Absolute values are applied to *p*-values. Note: Dashed arrows represent moderating effect, sold arrows represent direct relationships.

The results of the structural model, delineated in Table 4 and Figure 3, shed light on the hypothesized direct relationships between AMPs, JBO, PT, and PJF with WFW, accounting for 61%, 56.6%, and 18% of the variation, respectively. Notably, AMPs were found to exert a significant positive effect on job burnout (β = 0.390, *p* < 0.001), affirming H2. Conversely, no support was found for H1, as AMPs exhibited a non-significant impact on workforce well-being (β = −0.064, *p* = 0.339). Furthermore, strong direct relationships were observed between JBO and WFW (β = −0.154, *p* < 0.05), validating H3. Moreover, AMPs had a robust positive direct effect on perceived threat (β = 0.432, *p* < 0.001), supporting H4. PT exhibited a strong negative direct effect on WFW (β = −0.323, *p* < 0.001), corroborating H5.

### 4.5. Mediation Effect Analysis

To examine the mediating roles of JBO and PT in the relationship between AMPs and WFW, mediation analyses were conducted following the guidelines outlined by [86]. As shown in Table 5, JBO negatively and significantly mediated the relationship between AMPs and workforce well-being (β = −0.060, *p* < 0.05), providing support for H6. PT emerged as a significant mediator, with a negative and statistically significant indirect effect on WFW (β = −0.139, *p* < 0.001), thereby affirming H7. These findings highlight the multifaceted nature of the relationship between AMPs and workforce well-being, emphasizing the pivotal roles of JBO and PT as mediators in this dynamic interplay.

### 4.6. Analysis of the Moderating Effect of PJF

The examination of the moderating role of PJF in the relationship between AMPs and various outcome variables yielded insightful findings. Utilizing the product term by utilizing the PLS-SEM approach [87,88,89] and integrating PJF into the base model, interactions were explored to ascertain its influence on job burnout, perceived threat, and workforce well-being.

The statistical analysis, detailed in Table 5, revealed significant interactions between AMPs and PJF, affirming H8 and H9. Specifically, the product term of AMP x PJF exhibited significant positive correlations with both job burnout (β = 0.082, *p* < 0.010) and perceived threat (β = 0.104, *p* < 0.001), indicating that PJF positively moderated the relationships between AMPs and these adverse outcomes.

**Figure 4:**
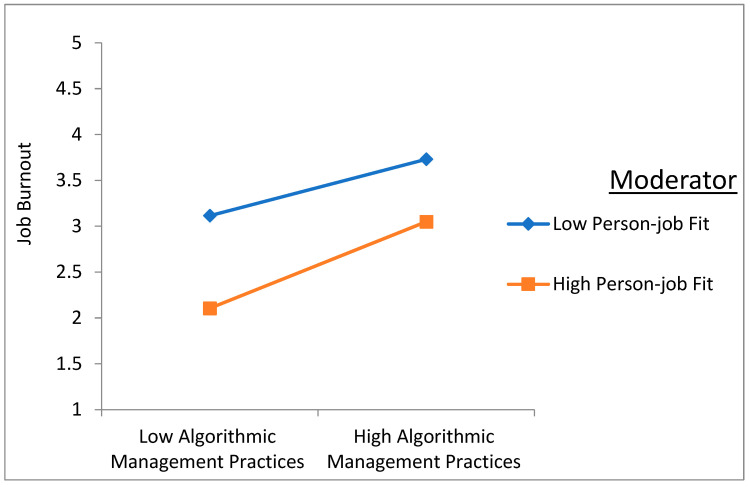
The moderation effect of PJF in the relationship between AMPs and job burnout.

**Figure 5:**
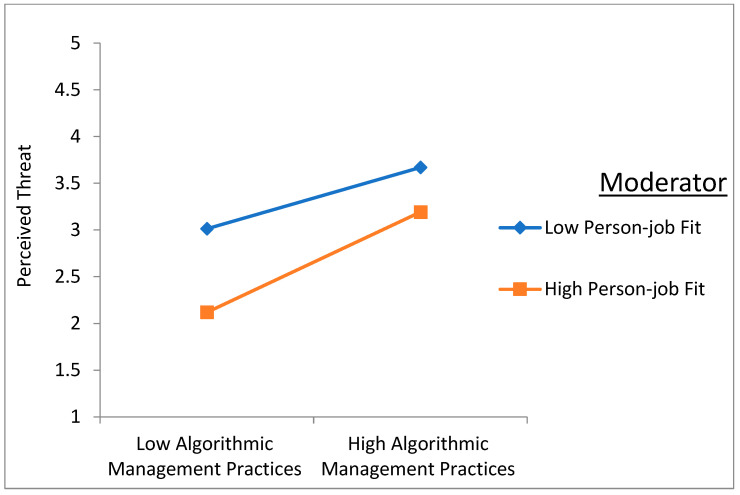
The moderation effect of PJF in the relationship between AMPs and perceived threat.

To elucidate the moderating effects further, simple slope analysis was employed, revealing nuanced insights into the relationship dynamics. As illustrated in Figure 4 and Figure 5, when PJF is high, the positive effects of AMPs on job burnout and perceived threat are amplified. Conversely, when PJF is low, the magnitude of the positive effects increases, indicating that PJF strengthens the adverse impact of AMPs on both job burnout and perceived threat.

**Figure 6:**
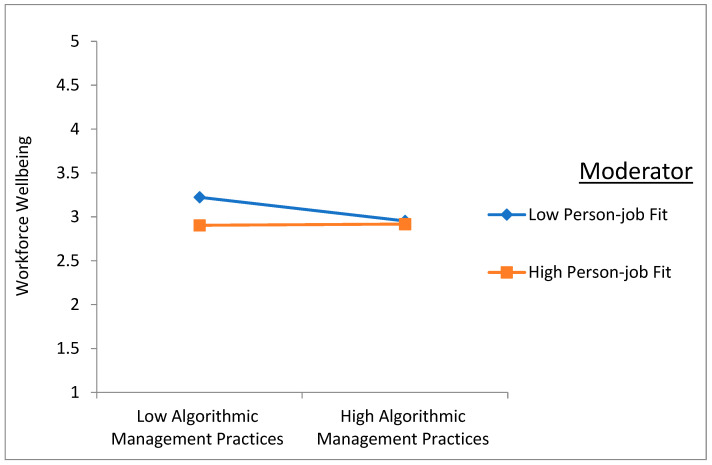
The moderation effect of PJF in the relationship between AMPs and workforce well-being.

However, contrary to expectations, the results pertaining to H10 did not provide support for the moderating effect of PJF on the relationship between AMPs and workforce well-being. The product term of AMP × PJF exhibited a marginally significant positive correlation with workforce well-being (β = 0.071, *p* < 0.100), suggesting a weak moderating influence of PJF. The simple slope analysis depicted in Figure 6 illustrated intersecting lines for low and high levels of PJF, indicating that PJF marginally dampens the negative relationship between AMPs and workforce well-being.

## 5. Discussion

This study investigates the complex relationships between algorithmic management practices, job burnout, perceived threat, workforce well-being, and the moderating role of person–job fit. The results provide valuable insights into how modern management practices influenced by technology can impact employees’ mental and emotional states and overall well-being. The results confirm that algorithmic management practices have a significant negative impact on workforce well-being. These practices often reduce employees’ autonomy, lead to constant surveillance, and create pressure to meet rigid performance metrics [90]. These factors collectively diminish job satisfaction, increase stress levels, and cause harm. The findings are consistent with self-determination theory, which emphasizes the importance of psychological well-being.

The analysis supports the hypothesis that algorithmic management practices contribute to higher levels of job burnout. The constant performance monitoring and pressure associated with algorithmic management practices exhaust employees’ emotional and physical reservoirs, resulting in fatigue and burnout. Also, [6] refers to the negative relationship between algorithmic management practices and workforce well-being. This aligns with conservation of resources theory, which asserts that stress arises from the risk of resource depletion or actual resource loss. The results confirm that job burnout substantially undermines workforce well-being. Job burnout, which is characterized by emotional fatigue and diminished professional effectiveness, directly influences employees’ mental and physical well-being, resulting in reduced job contentment and overall welfare [91].

The results indicate that algorithmic management practices heighten employees’ perceived threat. The impersonal nature of algorithms and the lack of human interaction can create feelings of job insecurity and anxiety about performance evaluations, contributing to a heightened sense of threat [92]. This research validates that perceived threat detrimentally influences workforce well-being. Employees facing perceived threats are prone to heightened stress and anxiety, resulting in adverse effects on their workforce well-being. The mediation analysis demonstrates that job burnout partially mediates the connection between algorithmic management practices and workforce well-being. While algorithmic management practices directly impact well-being, they channel a significant portion of this effect through the increased job burnout it causes [44].

Similarly, perceived threat and job burnout also mediate the relationship between algorithmic management practices and workforce well-being. Algorithmic management practices increase perceived threat, which in turn decreases workforce well-being. The findings emphasize the importance of addressing the psychological impacts of algorithmic management practices to mitigate their negative effects on well-being. Additionally, moderation analysis reveals that person–job fit weakens the positive relationship between algorithmic management practices and job burnout. Employees with a higher degree of person–job fit are better equipped to cope with the demands imposed by algorithmic management practices, experiencing less burnout compared to those with a lower degree of fit [50]. The findings also show that person–job fit moderates the relationship between algorithmic management practices and perceived threat. When employees have a higher degree of person–job fit, they are less likely to perceive algorithmic management practices as threatening, thereby reducing the negative impacts on their well-being.

Finally, person–job fit moderates the direct relationship between algorithmic management practices and workforce well-being [52,54]. Employees with a higher degree of person–job fit experience less negative impact on their well-being from algorithmic management practices compared to those with a lower degree of fit. This study highlights the significant negative impacts of algorithmic management practices on workforce well-being, primarily through increased job burnout and perceived threat. However, the presence of a positive person–job fit can mitigate these adverse effects, suggesting that organizations should consider the individual fit between employees and their roles when implementing algorithmic management practices. Ensuring that employees are well matched to their jobs can help alleviate some of the negative consequences of these modern management practices, ultimately supporting better workforce well-being. The integration of self-determination theory and conservation of resources theory provides a strong framework for understanding these dynamics and guiding future research and organizational strategies [19].

## 6. Conclusions

### 6.1. Theoretical Contribution

By examining the relationship between algorithmic management practices and workforce well-being, this research significantly advances our understanding of the intersection between technology-driven management and employee outcomes. Specifically, the integration of self-determination theory and conservation of resources theory offers a robust framework for analyzing how algorithmic management practices influence workforce well-being. Self-determination theory, which emphasizes the fulfillment of psychological needs such as autonomy, competence, and relatedness, helps explain how algorithmic management practices can undermine these needs, leading to decreased motivation and well-being among employees [93]. The rigid controls and constant monitoring associated with algorithmic management practices can diminish employees’ sense of autonomy and relatedness, resulting in lower job satisfaction and higher stress levels [16].

On the other hand, conservation of resources theory focuses on how individuals strive to obtain, retain, and protect resources, which are critical for their well-being. Algorithmic management practices’ potential to threaten or deplete these resources such as job security and personal agency can lead to heightened stress and job burnout, which negatively impacts workforce well-being [22]. This theoretical integration thus provides a comprehensive explanation for the mechanisms through which algorithmic management practices exert their effects on employees.

By examining the moderating role of person–job fit, this research sheds light on the conditions under which algorithmic management practices’ effects vary. The study shows that a higher degree of person–job fit can buffer the negative impacts of algorithmic management practices on employee outcomes by ensuring that job demands align with individual abilities, thereby reducing the stress and resource depletion that leads to job burnout [94]. This contribution is critical as it underscores the importance of considering individual differences and contextual factors in the application of algorithmic management practices, thereby enriching the literature on person–environment fit and employee well-being.

### 6.2. Practical and Managerial Implications

The findings of this study carry significant practical and managerial implications for organizations adopting algorithmic management practices. Given the adverse effects of algorithmic management practices on workforce well-being, organizations need to implement strategies that mitigate these negative outcomes. First, organizations should prioritize the integration of person–job fit assessments in their algorithmic management practices systems. Ensuring that employees are well-matched to their roles can reduce the likelihood of job burnout and perceived threat, as well as enhance overall employee satisfaction and well-being. As research suggests, a higher degree of person–job fit can buffer the adverse effects of algorithmic management practices, leading to better employee outcomes [94].

Moreover, transparency in algorithmic decision-making processes is crucial. Organizations should ensure that employees understand how decisions affecting their work are made and provide avenues for feedback and participation. This transparency can alleviate perceived threats and foster a sense of fairness and trust, which are critical for maintaining employee engagement and well-being [23].

Additionally, providing training and support tailored to the needs of employees working under algorithmic management practice systems can significantly improve their ability to cope with the demands of such environments. Training programs that enhance employees’ digital literacy and resilience can help them adapt to the technological changes imposed by algorithmic management practices, thus reducing job stress and improving workforce well-being [49]. Managers should be aware of the psychological impacts of algorithmic management practices and strive to create a work environment that supports autonomy, competence, and relatedness—key components of self-determination theory. By doing so, organizations can mitigate the dehumanizing effects of algorithmic management practices and promote a healthier, more supportive workplace [16].

### 6.3. Limitations and Future Direction

This research drew upon a distinct sample from a specific sector or entity, potentially constraining the broader applicability of the results. Subsequent studies should strive to validate these findings across varied samples to bolster their external relevance. The use of a cross-sectional framework limits our ability to infer causality regarding the investigated relationships. Future investigations could utilize longitudinal or experimental methodologies to establish causal connections and gain deeper insights into the temporal patterns of algorithmic management practices and employee consequences. While this study examined job burnout and perceived threat as mediating variables and person–job fit as a moderating variable, there may be other mechanisms at play. Future research could explore additional mediators and moderators to provide a more comprehensive understanding of the processes underlying the relationships examined in this study. This research predominantly focused on algorithmic management practices in a broad context, but the impacts may fluctuate depending on contextual variables such as organizational ethos, industry standards, and cultural norms. Subsequent investigations could delve into how these contextual elements shape the conservation of resources theory relations scrutinized in this study. By acknowledging these constraints and exploring avenues for future research, we can improve our understanding of the influence of algorithmic management practices on employee welfare and inform strategies for promoting positive outcomes in the workplace.

## Figures and Tables

**Table 1:** Characteristics of the sample.

Variables	Options	Frequency	Percentage (%)
Gender	Male	401	60.2%
	Female	265	39.8%
Organizational tenure	Less than 5 years	156	23.4%
	6–less than 10	206	30.9%
	11–less than 15	125	18.8%
	16–less than 20	110	16.5%
	More than 20 years	69	10.4%
Industry sectors	Food and Beverage	160	24.0%
	Electrical Equipment Manufacturing	115	17.3%
	Wood and Chemical Manufacturing	102	15.3%
	Machinery and Equipment Manufacturing	93	14.0%
	Rubber and Plastic Manufacturing	67	10.1%
	Textile and Clothing Manufacturing	55	8.3%
	Pharmaceuticals and Medical Equipment	43	6.4%
	Other Manufacturing	31	4.6%
Number of employees	Small-sized firms (less than 50 employees)	281	42.2%
	Medium-sized firms (less than 250 employees)	385	57.8%
Total		666	100%

**Table 2:** Validity and reliability of the measurement model.

Construct	Indicators	Mean	Std. Deviation	Outer Loadings	VIF	Cronbach’s α	CR	AVE
AMPs (AMP)						0.954	0.966	0.878
	AMP1	2.841	1.138	0.913	1.880			
	AMP2	2.785	1.043	0.956	3.476			
	AMP3	2.869	1.045	0.944	3.098			
	AMP4	2.859	1.015	0.935	2.824			
Job Burnout (JBO)						0.932	0.943	0.624
	JBO1	3.237	1.077	0.735	2.447			
	JBO2	3.050	1.035	0.725	2.914			
	JBO3	3.595	0.996	0.781	2.588			
	JBO4	3.290	1.022	0.732	2.590			
	JBO5	3.249	1.023	0.786	3.249			
	JBO6	3.123	1.035	0.825	3.723			
	JBO7	3.155	1.046	0.862	3.026			
	JBO8	3.258	1.029	0.859	4.386			
	JBO9	2.908	1.016	0.877	3.336			
	JBO10	2.940	1.016	0.690	2.679			
Perceived Threat (PT)						0.88	0.918	0.737
	PT1	3.197	1.114	0.872	2.685			
	PT2	3.215	1.261	0.832	1.987			
	PT3	3.002	1.186	0.898	3.046			
	PT4	3.279	1.084	0.831	1.960			
Person–job fit (PJF)						0.881	0.91	0.629
	PJF1	3.261	1.071	0.834	2.990			
	PJF2	3.243	1.070	0.866	3.691			
	PJF3	3.218	1.075	0.867	3.525			
	PJF4	2.821	1.094	0.678	1.467			
	PJF5	3.290	1.153	0.733	2.634			
	PJF6	3.360	1.125	0.760	2.689			
Workplace Well-Being (WFW)						0.879	0.901	0.532
	WFW1	2.548	1.201	0.680	2.234			
	WFW2	3.032	1.196	0.671	2.080			
	WFW3	2.629	1.246	0.695	3.007			
	WFW4	2.356	1.167	0.681	3.016			
	WFW5	3.425	1.088	0.730	1.928			
	WFW6	2.911	1.071	0.816	1.993			
	WFW7	3.428	1.022	0.744	2.411			
	WFW8	3.303	1.032	0.804	2.500			

Note(s): Variance inflation factor (VIF), composite reliability (CR), average variance extracted (AVE).

**Table 3:** Discriminant validity values.

Factors	1	2	3	4	5
1. AMPs (AMP)	***0.937***	*0.582*	*0.183*	*0.629*	*0.450*
2. Job Burnout (JBO)	0.730	***0.790***	*0.469*	*0.197*	*0.479*
3. Person–Job Fit (PJF)	−0.782	−0.736	***0.793***	*0.211*	*0.605*
4. Perceived Threat (PT)	0.712	0.729	−0.690	***0.858***	*0.225*
5. Workplace Well-Being (WFW)	−0.337	−0.364	0.300	−0.405	***0.729***

Note(s): Diagonals (in bold and italic) represent the square root of AVE, while the lower triangular pattern represents the shared variance (the squared correlations), and the upper triangular pattern (in italics) represents the HTMT correlation values.

**Table 4:** Estimates of direct effects.

Hypothesis	Hypothesized Relationships	Sample Estimate	Standard Error	T-Statistics	*p* Values	Decision
H1	AMP → WFW	−0.064 *^n.s^*	0.067	0.965	0.339	Not Supported
H2	AMP → JBO	0.390 ***	0.041	9.609	0.000	Supported
H3	JBO → WFW	−0.154 *	0.071	2.185	0.031	Supported
H4	AMP → PT	0.432 ***	0.045	9.590	0.000	Supported
H5	PT → WFW	−0.323 ***	0.066	4.790	0.000	Supported

Note(s): *n.s*, statistically non-significant relationships; *, statistically significant at *p* < 0.050; ***, statistically significant at *p* < 0.001.

**Table 5:** Indirect and interaction effect results.

Hypothesis	Hypothesized Relationships	Sample Estimate	Standard Error	T-Statistics	*p* Values	CIs		Decision
						0.025	0.975	
H6	AMP → JBO → WFW	−0.060 *	0.029	2.080	0.038	−0.117	−0.006	Supported
H7	AMP → PT → WFW	−0.139 ***	0.031	4.532	0.000	−0.206	−0.083	Supported
H8	AMP × PJF → JBO	0.082 **	0.024	3.375	0.001	0.034	0.130	Supported
H9	AMP × PJF → PT	0.104 ***	0.021	5.059	0.000	0.064	0.145	Supported
H10	AMP × PJF → WFW	0.071 †	0.042	1.705	0.088	−0.015	0.148	Not Supported

Note(s): †, statistically significant at *p* < 0.100; *, statistically significant at *p* < 0.050; **, statistically significant at *p* < 0.010; ***, statistically significant at *p* < 0.001; CI, confidence interval.

## Data Availability

The data from this study can be requested from the conservation of resources theory corresponding author, Ahmad Alzubi.

## Appendix A

**Table A1:** Measurement items.

**Algorithmic Management Practices [6,64]**
In this establishment, the pace of work of many employees is determined by machines or computers. This establishment use data analytics to monitor employee performance. This establishment use data analytics to improve the processes of production or service delivery. Robots carry complex series of actions automatically, which may include the interaction with people.
**Job Burnout [65,66]**
Every day I always get up without spirit, facing the work is listless.
2. I feel very tired after working all day.
3. My work often makes me feel bored.
4. I often need to consume too many emotional resources at work.
5. I feel confused about my current career prospects.
6. I have lost enthusiasm for my current job.
7. I think my current job is of little value.
8. I feel I’m not qualified for my current job.
9. Leaders often put me in charge of unimportant things.
10. I often have no confidence in the tasks assigned to me by the leader.
**Perceived Threat [68,69]**
The increased use of robots in our everyday life is causing more job loss for humans
2. In the long run, robots pose a direct threat to human safety and well-being
3. Recent advances in robot technology are challenging the essence of human life
4. Technological advancements in the robotic are threatening human’s uniqueness
**Person–Job Fit [70]**
The experience and skills required to operate service robots match my own experience, skills, and knowledge.
2. I believe my skills and abilities match those required by the job.
3. My job performance is hurt by a lack ofexpertise on the job. (R)
4. I feel that this work enables me to do the kind of workI want to do.
5. This work is a good match for me.
6. This work is the type that I’m seeking.
**Workplace Well-Being [71]**
In the past 6 months,
In my work, I achieve my potential.
2. In my work, I develop abilities that I consider important.
3. In my work, I engage in activities that express my skills.
4. In my work, I overcome challenges.
5. In my work, I achieve results that I regard as valuable.
6. In my work, I advance in the goals I set for my life.
7. In my work, I do what I really like doing.
8. In my work, I express what is best in me.
